# Defining routine fatigue care in Multiple Sclerosis in the United Kingdom: What treatments are offered and who gets them?

**DOI:** 10.1177/20552173211072274

**Published:** 2022-01-20

**Authors:** Federica Picariello, Jennifer Freeman, Rona Moss-Morris

**Affiliations:** School of Health Professions, Faculty of Health, 6633 University of Plymouth, Plymouth, UK of Great Britain and Northern Ireland; Health Psychology Section, Institute of Psychiatry, Psychology and Neuroscience, 4616 King's College London, London, UK of Great Britain and Northern Ireland

**Keywords:** Fatigue, multiple sclerosis, UKMSR, implementation, behavioural interventions, exercise interventions, Amantadine, routine care

## Abstract

**Background:**

Fatigue is common and disabling in Multiple Sclerosis (MS). A recent meta-analytic systematic review reported 113 trials of exercise and behavioural interventions for fatigue, yet patients consistently describe fatigue being under-treated. The extent of the research-to-practice gap is yet to be documented.

**Objective:**

To describe what fatigue treatments people with MS (pwMS) in the United Kingdom (UK) have been offered.

**Methods:**

A cross-sectional survey of pwMS on the UK MS Register (UKMSR). Data on fatigue treatments offered were collected using an online questionnaire developed with patient input and summarised using descriptive statistics. Sociodemographic, MS-related, and psychological factors associated with treatment offered were evaluated using a logistic regression model.

**Results:**

4,367 respondents completed the survey, 90.3% reported experiencing fatigue. Of these, 30.8% reported having been offered at least one type of pharmacological/non-pharmacological treatment for fatigue. Pharmacological treatments were more commonly offered (22.4%) compared to non-pharmacological treatments (12.6%; 2.9% exercise and 5.9% behavioural therapy). In the logistic regression model, older age, working, shorter time since MS diagnosis, and lower fatigue were associated with lower odds of having been offered treatment for fatigue.

**Conclusion:**

This study accentuates the extent of the unmet need for fatigue treatment in MS in the UK.

## Background

Fatigue, extreme and persistent tiredness/exhaustion with decreased physical and/or mental capacity, not linked to energy expenditure, and unalleviated by rest;^1^ is one of the most prevalent and disabling symptoms of Multiple Sclerosis (MS).^2^ Estimates of fatigue prevalence range between 50–80%^2–4^ with a recent survey using the national MS Register (UKMSR) showing that 56% of people with MS (pwMS) report severe fatigue.^5^ Fatigue has high costs to the individual,^6,7^ society and the National Health Service (NHS).^8^

The National Institute for Health and Care Excellence (NICE) guidelines recommend Amantadine, exercise, and behavioural interventions for MS-fatigue.^9^ Systematic reviews suggest pharmacological treatments for fatigue, including Amantadine, display limited efficacy and are inferior to exercise and behavioural interventions.^10^ Yet, in the NICE guidelines, Amantadine is presented as the treatment of choice, with behavioural and exercise interventions considered alternative or adjunctive treatment options with limited directive on these.^9^ Two systematic reviews of exercise and behavioural trials, including a meta-analytic review of interventions designed specifically for MS-fatigue (n = 34)^11^ and network meta-analysis (n = 113)^12^ of all MS trials with fatigue as an outcome, found cognitive-behavioural therapy (CBT) and exercise interventions focused on improving balance or including a combination of aerobic, strength, balance, flexibility, performed better than other interventions. Energy conservation which is offered as a treatment to pwMS demonstrated minimal effectiveness.^11,12^ Despite the large number of different interventions evaluated and positive findings for a core of these, a 2011 national audit of services for pwMS showed fatigue was consistently reported to be a major concern but not well treated.^13^ What exactly is currently offered as routine treatment for MS fatigue in the UK remains unclear.

One behavioural intervention, FACETS, a group fatigue self-management intervention that combines CBT and energy conservation^14,15^ has been adopted by some services. By 2015, FACETS was estimated to have been delivered to 1,500 pwMS in the UK with 200 healthcare professionals (HCPs) trained to deliver it.^16^ Given that at least 71,120 pwMS are likely to experience severe fatigue,^5^ 1,500 represents 2% of the target population. Such implementation data are unfortunately not available for other fatigue treatments. Of note, there was no end-of-treatment treatment effect of FACETS and only a small effect was evident one year later.^14,15^ Groups may also be challenging for pwMS with work commitments, mobility or cognitive impairments, rurality and transport issues to attend. Larger treatment effects have been observed for other exercise and behavioural interventions, but few pragmatic trials have been conducted.^11,12^ Importantly, no specific evidence is currently available on disparities in access to fatigue treatments.

The aim of this study was to gain a more detailed picture of how MS fatigue is currently addressed by NHS services in the UK through a survey of pwMS on the UK MS Register (UKMSR) with the following objectives:

- To estimate the number of people experiencing fatigue as an MS symptom
- To compare the sociodemographic, MS-related, and psychological characteristics between pwMS reporting fatigue and those not reporting fatigue
- To describe what treatments pwMS had been previously offered for fatigue
- To describe level of perceived improvement in fatigue following treatment
- To identify sociodemographic, MS-related, and psychological factors associated with having been offered treatment for fatigue

## Methods

### Design

This was a cross-sectional online survey of pwMS^1^.

### Sample

Adults with a confirmed MS diagnosis in the UK have been able to enrol to the UKMSR (https://www.ukmsregister.org/) since May 2011. Currently, the UKMSR has more than 16,000 consented patients of whom 3,148 are considered active defined as completing one of the core questionnaires in the last 2 data collection windows. The UKMSR sample is representative of the MS population more generally.^17^ UKMSR's recruitment strategy consists of 1) advertising through social media, radio, TV, magazines, large MS-related events/conferences and 2) NHS partner services. Clinical data are collected by the individual taking consent, such as a nurse or neurologist, while self-reported demographic, clinical, and outcome data are collected online supplied directly by the respondents. Online questionnaires are sent out to users every six months, also prompting respondents to update their basic sociodemographic and clinical characteristics.

### Data collection

The following data were extracted from the UKMSR: age, gender, ethnicity, employment status, education status, time since MS diagnosis, MS subtype, and the web-based Expanded Disability Status Scale (EDSS)^18^ - the standard criterion for assessing disability in MS. Fatigue, depression and anxiety, walking ability, and physical and psychological impact of MS are core patient-reported outcomes collected routinely by the UKMSR (Supplementary file A) and were also extracted. Scores closest to the current data collection window were obtained.

### Fatigue treatments offered

With input from pwMS, consisting of an informal discussion in a large meeting, feedback on draft version from nine pwMS, and further feedback on a revised demo version from four pwMS, we developed a questionnaire to capture what treatments, if any, pwMS had been previously offered for fatigue (Supplementary file B for content overview). Participants were first asked to indicate whether they had ever experienced fatigue as a symptom of their MS; only those who reported ever experiencing fatigue were invited to complete the questions related to treatments they had been offered (from here onwards referred to as ‘fatigue treatment(s) offered’. The questionnaire was deployed to all consented pwMS (n = 16,000) registered on the UKMSR between March and April 2021.

### Statistical analysis

Statistical analyses were performed using SPSS v21 (SPSS Inc., Chicago, IL). For the standardised questionnaires, the overall score was calculated. Item-level missingness was negligible across these questionnaires (1 incomplete HADS response and 1 incomplete MSIS-29 response) so available-case analysis was adopted. Participants were included in the analyses if they completed the fatigue treatments questionnaire. Participants reporting ever experiencing fatigue as a symptom of their MS versus those not reporting fatigue were compared using independent-measures t-tests for continuous data, Chi-square for categorical data or Fischer's exact alternative where expected cell count was below 5 in more than 20% of cells. Data on fatigue treatments offered were summarised using descriptive statistics.

Univariate logistic regressions were calculated to assess the relationships between sociodemographic, MS-related, and psychological variables and fatigue treatment offered (any fatigue treatment, at least one pharmacological treatment, at least one non-pharmacological treatment for fatigue, at least one exercise treatment for fatigue, or at least one behavioural treatment for fatigue). Due to very low numbers of pwMS from BAME backgrounds, a crude dichotomous categorisation for ethnicity was used in the analyses: white and BAME. Employment status was also recoded into the following categories: working, not working, and retired due to scarcity of data across some of the categories. Age, gender, and ethnicity, as well as variables which showed a significant relationship in univariate analysis were entered into a final multivariate logistic regression model. The low event rate across treatment subtypes precluded multivariate analyses using treatment subtypes as dependent variables. Disability status based on the web-EDSS was only available in 21% of the sample and for this reason was not used in multivariate analysis.

Qualitative data arising from free text responses were analysed by creating units of analysis from each response, a phrase containing only one concept, and sorting these units of analysis into categories by meaning.

### Ethical approval

The UKMSR has been approved as a study by the South West Central Bristol Research Ethics Council (initial registration code 11/SW/0160; renewed registration code 16/SW/0194).^17,19^ In line with procedures, approval for this specific study was obtained from the internal ethical committee of the UKMSR. Written informed consent for data to be used for research projects is provided by all pwMS on enrolment in UKMSR. Data access was fully anonymised.

## Results

4,367 pwMS accessed the fatigue treatments offered questionnaire and could be included in the analysis, which represents 139.7% of the 3,148 users defined as active. Considering users who were active during the same live data collection window specifically (n = 4,693), the response rate was 93.1%.

### Sample characteristics

Table 1 provides an overview of the sample characteristics. The sample was predominantly female (74.2%), of White ethnic origin (93.1%), in regular paid employment (35.5%) or retired (31.0%), with a mean age of 55.13 years (SD = 11.29). RRMS was the most common MS subtype (56.2%). Disability status based on web-based EDSS was available only in 21% of the sample, with a mean of 5.01 (SD = 2.07) indicative of some loss of ambulatory ability. Based on the MSWS, 28.3% of the sample reported being unable to walk. The mean time since MS diagnosis was 19.18 years (SD = 11.83), with a wide range. The mean fatigue severity score based on the averaged FSS was 4.83 (SD = 1.52) and 63.0% of respondents were experiencing severe fatigue based on a cut-off of 5 on the FSS.^20^

**Table 1.** Characteristics of survey respondents (N = 4367).

Variable	Statistic
Age (n = 4367)	55.13 (SD = 11.29; range = 20-87)
Gender (n = 4367)	
Female	3242 (74.2%)
Male	1122 (25.7%)
Prefer not to say	3 (0.1%)
Ethnicity (n = 4367)	
White	4066 (93.1%)
Black	30 (0.7%)
Asian	46 (1.1%)
Mixed	27 (0.6%)
Other	180 (4.1%)
Missing	18 (0.4%)
Education status (n = 4367)	
Primary school	1 (0.0%)
Secondary school	834 (19.1%)
Occupational certificate or diploma	1318 (30.2%)
Undergraduate degree	1131 (25.9%)
Postgraduate degree	833 (19.1%)
Other	250 (5.7%)
Employment status (n = 4367)	
Regular paid employment	1552 (35.5%)
Self-employed	277 (6.3%)
Engaged in voluntary work	55 (1.3%)
In formal education	24 (0.5%)
Looking after home/family	127 (2.9%)
Retired	1353 (31.0%)
Unemployed	94 (2.2%)
Temporarily sick/disabled	51 (1.2%)
Permanently sick/disabled	776 (17.8%)
Other reasons not working	40 (0.9%)
Not applicable	17 (0.4%)
Missing	1 (0.0%)
MS subtypes (n = 4367)	
RRMS	2454 (56.2%)
SPMS	1109 (25.4%)
PPMS	521 (11.9%)
Benign	96 (2.2%)
Unknown	187 (4.3%)
Time since MS diagnosis in years (n = 4162)	19.18 (SD = 11.83; range = 0-66)
Web-based EDSS (n = 919)	5.01 (SD = 2.07; range = 0-9.50)
Fatigue severity (FSS total score) (n = 4238)	43.50 (SD = 13.66; range = 9-63)
Fatigue severity (FSS average score) (n= 4238)	4.83 (SD = 1.52; range = 1-7)
Fatigue status based on FSS (n = 4238)	
Severe fatigue (≥5 FSS score)	2671 (63.0%)
Walking impairment (MSWS) (n = 2994)	39.02 (SD = 31.76; range = 0-100)
Able or not able to walk (MSWS) (n = 4367)	
Able to walk	2994 (68.6%)
Unable to walk	1235 (28.3%)
Missing	138 (3.2%)
Distress (HADS) (n = 4217)	13.32 (SD = 8.26; range = 0-42)
MS Impact - Physical (MSIS-P) (n = 4233)	32.08 (SD = 20.29; range = 0-75)
MS Impact - Mental (MSIS-M) (n = 4233)	28.14 (SD = 18.21; range 0-75)

*Note.* RRMS = Relapsing-Remitting MS; SPMS = Secondary Progressive MS; PPMS = Primary Progressive MS

### Differences between respondents experiencing fatigue and those not experiencing fatigue

Out of the respondents, 90.3% reported experiencing fatigue as a symptom of their MS. Significant differences were evident between respondents reporting fatigue and those not reporting fatigue. Those reporting fatigue as a symptom of their MS were more likely to have lower level of education (p = 0.006), less likely to be in regular paid employment and more likely to be temporarily or permanently sick/disabled (p < 0.001), more likely to have SPMS and less likely to have benign MS (p < 0.001), with a longer duration of MS (p < 0.001), higher levels of disability (p < 0.001), higher levels of fatigue severity (p < 0.001), walking impairment (p < 0.001), distress (p < 0.001), and physical and mental impact of MS (p < 0.001). Supplementary file C details all the comparisons.

All remaining analyses are conducted on those who reported fatigue only (n = 3943).

### Fatigue treatments offered

**Figure 1.**
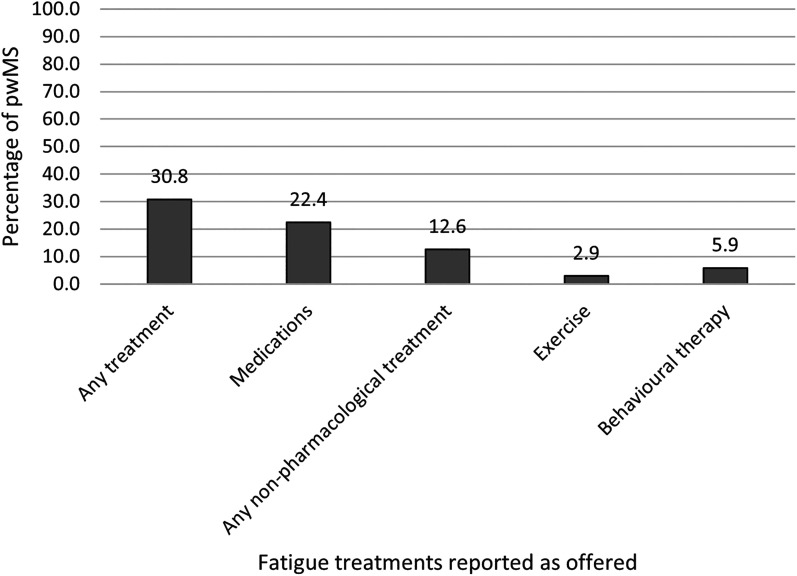
Fatigue treatments reported as offered (n = 3943).

Figure 1 summarises types of fatigue treatment(s) offered. 30.8% (n = 1214) had been offered at least one type of treatment for fatigue. Pharmacological treatments were more commonly reported (22.4%, n = 883) compared to non-pharmacological treatments (12.6%, n = 498; see Figure 1). 18.2% (n = 716) reported having been offered exclusively pharmacological treatment(s) for fatigue, while 8.4% (n = 331) exclusively non-pharmacological treatments for fatigue. Only a minority of respondents reported having been offered both (4.2%, n = 167). 83.5% (n = 3292) wanted better provision of fatigue treatments.

A detailed breakdown by type of treatment is provided in Table 2 and outlined below.

**Table 2.** Fatigue treatments offered by subtype*.

	
*MEDICATIONS*	
Types of medications	
Amantadine	447 (50.6%)
Modafinil	282 (31.9%)
Prokarin	1 (0.1%)
Other prescription medications	69 (7.8%)
Over the counter medications (such as liquid iron, beetroot powder, vitamin B12)	175 (19.8%)
Do not recall name of medication	50 (5.7%)
*EXERCISE*	
Exercise	
No	2778 (70.5%)
Yes, but not for fatigue specifically	1050 (26.6%)
Yes, for fatigue specifically	115 (2.9%)
Types of exercise offered	
Aerobic	14 (12.2%)
Resistance	29 (25.2%)
Yoga	17 (14.8%)
Balance	58 (50.4%)
Other	17 (14.8%)
Physiotherapy	54 (47.0%)
*BEHAVIOURAL THERAPY*	
Behavioural therapy	
No	3594 (91.1%)
Yes, but not for fatigue specifically	118 (3.0%)
Yes, for fatigue specifically	231 (5.9%)
Types of behavioural therapy offered	
Counselling	32 (13.9%)
CBT	29 (12.6%)
Mindfulness	45 (19.5%)
FACETS	127 (55.0%)
Not sure	46 (20.0%)
Other	12 (5.2%)
*DIETITIAN SUPPORT*	
Based on free text responses, dietitian support consisted of advice related to meal organisation and specific foods that may boost or hinder energy levels.	
Dietitian support	
No	3696 (93.7%)
Yes, but not for fatigue specifically	244 (6.2%)
Yes, for fatigue specifically	3 (0.1%)
*NURSE SUPPORT*	
Based on free text responses, nurse support mostly consisted of general advice and support, fatigue management course recommendations, or referral to physiotherapy or behavioural therapy. Most commonly, advice centred on energy conservation and pacing: “*An analogy of a mobile phone. Don't let the battery go too low as it takes a lot longer to fully charge. Charge when quite full”.* Respondents also reported being told to rest when tired as a fatigue management strategy.	
Nurse support	
No	2007 (50.9%)
Yes, but not for fatigue specifically	1791 (45.4%)
Yes, for fatigue specifically	145 (3.7%)
*SOCIAL CARE SUPPORT*	
Based on free text responses, social care support predominantly consisted of provision of equipment, such as perching stool, and personal assistance, such as someone providing support with housework. The issue of funding for personal assistance was raised by respondents: “*As a single parent I had a personal assistant to help me cope. I stopped it as I couldn't afford it. But it was invaluable help*”.	
Social care support	
No	3661 (92.8%)
Yes, but not for fatigue specifically	258 (6.5%)
Yes, for fatigue specifically	24 (0.6%)
*OCCUPATIONAL THERAPY SUPPORT*	
Based on free text responses, needs assessment at home and/or work was a central feature of occupational therapy support, for instance: “*At work (supermarket) I changed my job role to an admin-based one, and also now use a trolley to carry price tickets around, and leaning on the trolley whilst moving around helps a lot through the day*”. Provision of equipment, such as a perching stool, to facilitate energy-saving was also a recurrent feature of occupational therapy support. Similarly to nurse support, advice for fatigue management was predominantly geared to energy conservation and pacing: “*An occupational therapist helped me to map out energy usage and make plans to pace myself*” and “*I saw an occupational therapist…to help ‘manage my fatigue’. Which is a phrase that seems to me more and more to mean ‘learn to put up with doing less’*”.	
Occupational therapy support	
No	3097 (78.5%)
Yes, but not for fatigue specifically	724 (18.4%)
Yes, for fatigue specifically	122 (3.1%)

*Note.* %s for each treatment subtype may exceed 100% as multiple options could be selected.

#### Pharmacological treatments

**Figure 2.**
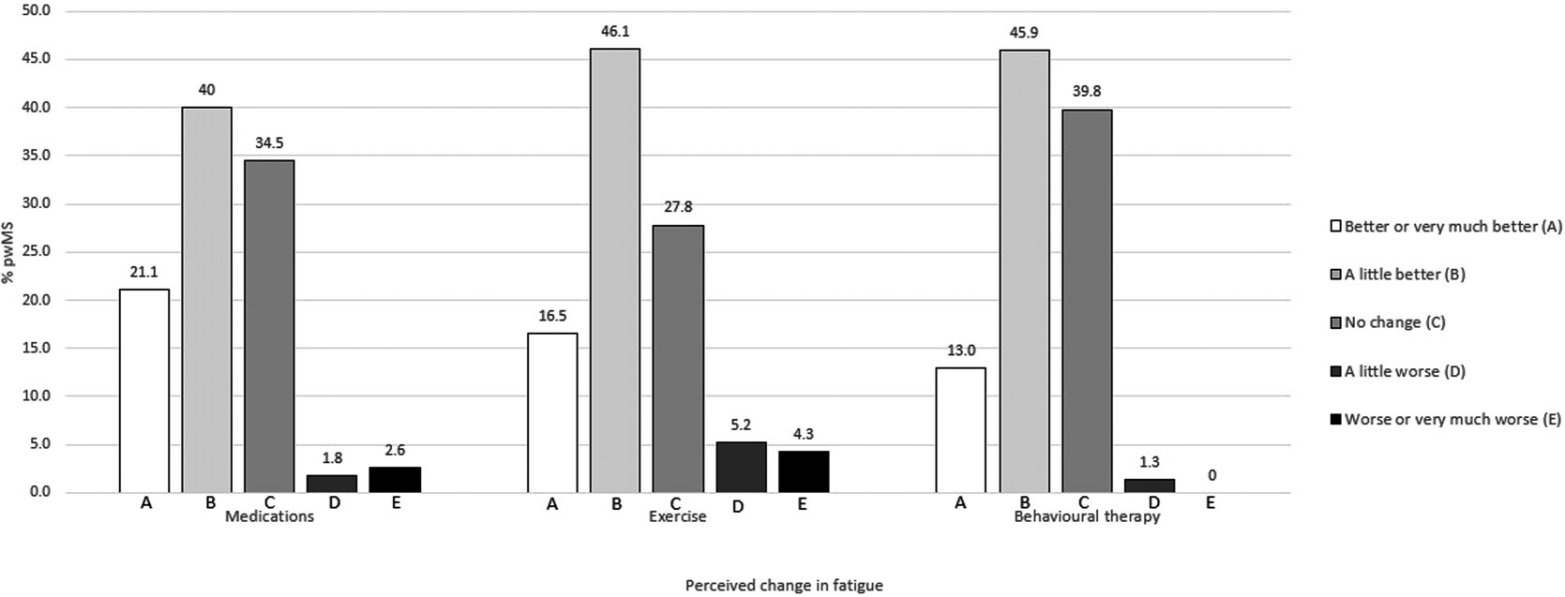
Perceived change in fatigue following medications or exercise or behavioural therapy.

77.6% (n = 3069) had not been offered any medications for fatigue. Of those reporting being offered medication, the majority reported having been offered only one type of medication (86.2%, n = 761), the most common being Amantadine. The median perceived change in fatigue following medication was 3 (interquartile range = 1, range = 1–7) indicative of some improvement, with 61.1% reporting some level of improvement (Figure 2).

In the free text responses, the side-effects of Amantadine and Modafinil were commonly raised by respondents: “*I had Amantadine but it left me feeling like I was swimming through mud mentally*” and “*I take Modafinil which helps quite a bit, but also makes me feel a bit strange (like speedy)…it makes me more tired the day after I’ve taken it*”.

Other medications mentioned by respondents were low-dose Naltrexone, anti-depressants, such as Amitriptyline, and medications targeting other symptoms like pain, spasticity, and difficulties with walking which were described to have secondary benefits for fatigue: *“I tried Fampridine to assist with walking. It helped with walking, but I also found that it helped with fatigue*”.

#### Exercise

2.9% (n = 115) respondents reported having been offered at least one type of exercise therapy specifically for their fatigue, with balance exercise or physiotherapy the most common. In the free text responses, dance and swimming were also mentioned. Of those reporting being offered exercise, most had been offered one or two types of exercise therapy (50.4%, 37.4%, respectively), with only a handful having been offered 3 or 4 types of exercise therapy (12.2%, n = 14). The median perceived change in fatigue following exercise was 3 (interquartile range = 1, range = 1–7) indicative of some improvement, with 62.6% reporting some level of improvement (Figure 2).

#### Behavioural therapy

5.9% (n = 231) respondents reported having been offered at least one type of behavioural therapy specifically for their fatigue, with the most common offered being FACETS. In the free text responses, additional fatigue management courses delivered in a group format were mentioned, but only some specific to MS. Of those reporting being offered behavioural therapy, most had been offered one type of therapy (78.8%, n = 182). The median perceived change in fatigue following behavioural therapy was 3 (interquartile range = 1, range = 1–5) indicative of some improvement, with 58.9% reporting some level of improvement (Figure 2).

#### Other non-pharmacological treatments

Only a minority reported having been offered dietitian, nurse, social care or occupational therapy support for fatigue specifically (0.1%, 3.7%, 0.6%, 3.1%, respectively). Free text responses provided further detail on these support options (see Table 2). Perceived change in fatigue following these support options was generally indicative of little to no change in fatigue (Supplementary file D).

### Univariate associations between sociodemographic, MS-related, and psychological variables and fatigue treatment offered receipt

In univariate logistic analysis, employment status (not employed), longer time since MS diagnosis, and MS subtype (SPMS) were associated with having been offered treatment(s) for fatigue (Table 3). In terms of self-report questionnaires, higher disability (web-based EDSS), fatigue, distress, physical and psychological impact of MS, and walking impairment were associated with having been offered treatment(s) for fatigue. Patterns of univariate associations were generally similar when looking at subtypes of fatigue treatment offered. However, these coefficients need to be treated with caution given the very low event rate when looking at treatment subtypes.

**Table 3.** Univariate associations between sociodemographic, MS-related, and psychological variables and fatigue treatments offered.

	Any treatment offered		Pharmacological treatment offered^†^		Non-pharmacological treatment offered^†^		Exercise offered^†^		Behavioural therapy offered^†^	
	OR	95% CI	OR	95% CI	OR	95% CI	OR	95% CI	OR	95% CI
Age	0.99	0.99-1.00	1.00	0.99-1.00	0.99	0.99-1.00	1.01	1.00-1.03	0.99	0.98-1.01
Gender (Female vs. Male)	0.96	0.82-1.12	0.94	0.79-1.12	1.01	0.81-1.25	0.49*	0.33-0.71	1.30	0.94-1.80
Ethnicity (BAME vs. White)	1.24	0.95-1.63	1.02	0.75-1.39	1.50*	1.06-2.11	1.12	0.54-2.32	1.20	0.72-2.00
Education status										
Occupational certificate/diploma vs. school	1.13	0.93-1.37	1.16	0.93-1.44	1.30	0.97-1.73	2.03*	1.10-.3.72	1.26	0.83-1.93
Undergraduate vs. school	1.15	0.94-1.41	1.08	0.87-1.36	1.41*	1.05-1.89	1.92*	1.02-3.59	1.54*	1.01-2.36
Postgraduate vs. school	0.98	0.78-1.22	0.90	0.70-1.16	1.26	0.92-1.73	1.21	0.59-2.50	1.52	0.97-2.39
Other vs. school	0.98	0.71-1.36	1.02	0.71-1.46	1.24	0.79-1.97	1.48	0.56-3.89	1.44	0.76-2.73
Employment status										
Retired vs. working	1.24*	1.05-1.47	1.29*	1.07-1.55	1.12	0.89-1.41	2.05*	1.24-3.38	1.11	0.79-1.56
Not working vs. working	1.84**	1.55-2.19	1.87**	1.55-2.27	1.59**	1.26-2.01	2.70**	1.64-4.46	1.70*	1.22-2.36
Other^‡^ vs. working	1.50*	1.10-2.04	1.27	0.89-1.80	1.31	0.85-2.00	2.78*	1.28-6.02	1.56	0.88-2.77
Time since diagnosis	1.01**	1.01-1.02	1.02**	1.01-1.03	1.00	0.99-1.00	1.00	0.98-1.02	1.00	0.98-1.01
MS subtype										
SPMS vs. RRMS	1.27*	1.08-1.48	1.48*	1.25-1.75	0.92	0.74-1.15	1.19	0.76-1.85	0.86	0.63-1.20
PPMS vs. RRMS	1.01	0.82-1.26	0.78	0.60-1.01	1.27	0.96-1.67	1.99*	1.21-3.28	1.29	0.88-1.89
Benign vs. RRMS	0.54*	0.30-0.98	0.71	0.38-1.33	0.28*	0.09-0.90	0.52	0.07-3.84	0.43	0.10-1.76
Unknown vs. RRMS	0.85	0.59-1.21	0.92	0.62-1.37	0.67	0.39-1.15	1.20	0.47-3.03	0.58	0.25-1.34
Web-based EDSS	1.10*	1.02-1.18	1.12*	1.02-1.22	1.03	0.93-1.14	1.04	0.86-1.26	0.95	0.83-1.10
FSS	1.61**	1.51-1.71	1.60**	1.49-1.71	1.52**	1.39-1.65	1.77**	1.47-2.12	1.45**	1.29-1.63
HADS	1.04**	1.03-1.05	1.05**	1.04-1.06	1.02**	1.01-1.03	1.03*	1.01-1.05	1.01	1.00-1.03
MSIS-Physical	1.02**	1.02-1.02	1.02**	1.02-1.03	1.01**	1.01-1.02	1.02**	1.01-1.03	1.01*	1.00-1.02
MSIS-Mental	1.02**	1.02-1.03	1.02**	1.02-1.03	1.01**	1.01-1.02	1.01*	1.00-1.02	1.01*	1.00-1.02
Not able to walk based on MSWS	1.23*	1.06-1.43	1.33**	1.13-1.56	1.03	0.84-1.27	1.04	0.69-1.55	0.75	0.55-1.02
MSWS	1.01**	1.01-1.01	1.01**	1.01-1.01	1.01**	1.01-1.01	1.02**	1.01-1.03	1.01*	1.00-1.01

^*^ p < 0.05.

^**^ p < 0.001.

^†^ Low event rate (22.4%, 12.6%, 2.9%, and 5.9%, respectively) therefore estimates should be interpreted with caution.

^‡^ Volunteering, in education, taking care of family/home, and not applicable.

### Multivariate logistic model of fatigue treatment offered

In the multivariate analysis (Table 4), those working were less likely to have been offered treatment for fatigue in comparison to all other employment status categories. Those younger (OR = 0.98, 95% CI 0.97–0.99), with a longer time since MS diagnosis (OR = 1.02, 95% CI 1.01–1.03), and higher fatigue (OR = 1.52, 95% CI 1.40–1.65) were more likely to have been offered fatigue treatment. These variables explained 12% of the total variance (Nagelkerke R^2^).

**Table 4.** Multivariate regression predicting having been offered any treatment for fatigue (N = 3584).

	Any treatment offered	
	OR	95% CI
Age	0.98**	0.97-0.99
Gender (Female vs. Male)	0.89	0.75-1.06
Ethnicity (BAME vs. White)	1.32	0.98-1.81
Employment status		
Retired vs. working	1.38*	1.09-1.75
Not working vs. working	1.34*	1.08-1.66
Other^†^ vs. working	1.40^∼^	0.99-1.98
Time since diagnosis	1.02**	1.01-1.03
MS subtype		
SPMS vs. RRMS	0.98	0.79-1.22
PPMS vs. RRMS	1.05	0.80-1.39
Benign vs. RRMS	0.72	0.38-1.36
Unknown vs. RRMS	0.70	0.47-1.05
FSS	1.52**	1.40-1.65
MSIS-Physical	1.00	0.99-1.01
MSIS-Mental	1.01	1.00-1.02
Not able to walk based on MSWS	0.89	0.73-1.08

*Note.* HADS excluded from model due to high correlation with MSIS-Mental (r = 0.82, p < 0.001) and multicollinearity in the model.

^*^ p < 0.05.

^**^ p < 0.001.

^∼^ approaching significance.

^†^ Volunteering, in education, taking care of family/home, and not applicable.

## Discussion

### Overview of findings

To our knowledge, this is the first study to gather information on what routine care for fatigue consists of in the UK from the perspective of people with MS. The results of this large survey confirmed anecdotal reports that fatigue in MS is severely undertreated. 90% of pwMS reported experiencing fatigue as a symptom of their MS in the survey, in line with previous prevalence estimates,^2–5^ yet less than a third reported having been offered a fatigue treatment. The vast majority (83.5%) wanted better provision of fatigue treatments. Pharmacological treatments were most reported (22.4%), particularly Amantadine, while exercise or behavioural treatments for fatigue were seldom offered (2.9% and 5.9%, respectively). Balance exercise and physiotherapy exercise programmes were most reported for the category of exercise and FACETS in the category of behavioural treatments. Only a minority of respondents reported having been offered both pharmacological and non-pharmacological treatments for fatigue (4.2%). Notably, of those reporting being offered any treatment for fatigue (30.8%), at least some level of fatigue improvement following medications, exercise or behavioural treatments was reported among slightly more than half of pwMS (59% to 62%).

Those younger, those with a longer duration of MS, those not in regular employment, and those with higher levels of fatigue were more likely to have been offered some form of fatigue treatment in the multivariate regression model. It is interesting that being in regular employment was associated with lower likelihood of being offered fatigue treatment. This may indicate issues with practicality of attending treatments for pwMS who work, or lower levels of disability among those still able to work. Higher disability was associated with higher likelihood of being offered treatment in the univariate analysis. Fatigue is one of the main reasons pwMS reduce their work hours or retire early from work,^6^ therefore, treating fatigue earlier is arguably a priority, which requires flexibility for pwMS to integrate treatment into their daily lives.

### Previous evidence

The findings of this study concur with previous evidence on the unmet need for supportive therapies more generally in MS,^21^ although to date no study has specifically examined the range of treatments routinely offered for fatigue and factors that are associated with being offered treatment. Inadequate screening for and management of fatigue in clinical care has been previously described by patients across long-term conditions.^22,23^ In addition to lack of routine fatigue screening, HCPs may be unaware or uncertain of the best evidence-based approaches for the management of fatigue, particularly where a clear and defined treatment pathway does not exist.^24^ As previously raised, in the NICE guidelines non-pharmacological treatment recommendations for MS fatigue are poorly defined and there is lack of clarity in the guidelines as to which treatments are most effective.^9^ The findings also accentuate the research-to-practice gap as the treatments offered may not necessarily be the evidence-based ones, as observed with CBT that was less frequently reported compared to FACETS, mindfulness, and counselling, despite the promising evidence.^12^

Importantly, open-ended responses on pharmacological treatments also revealed the complex and intricate inter-relationships between symptoms of MS, highlighting that fatigue is unlikely to occur in isolation. Relatedly, in a recent study in kidney failure, symptom clustering was observed,^25^ with some evidence of symptom clusters emerging in MS as well.^26,27^ It may therefore be important for the inter-connectedness of symptoms to be acknowledged as part of fatigue treatment, advocating for a more tailored and needs-based approach.^28^ For example, in our network meta-analysis^12^ favourable evidence was observed for different subtypes of exercise which indicates that there may not be a single optimal exercise modality and the mechanisms through which exercise improves fatigue likely differ between different exercise interventions, which may in part be determined by the combination of symptoms experienced.^29^

### Clinical implications

Although the number of trials evaluating fatigue interventions continues to grow, a research-to-practice gap is evident based on the findings here. The lag between research and implementation is well documented, with a landmark study estimating that it takes 17 years to turn 14% of research into practice and patient benefit.^30^ A number of reasons are likely to be responsible for the research-to-practice gap. The majority of interventions are not developed with the context of delivery in mind, meaning that they may rely on staff or resources that are rarely available in routine care or require a time commitment that would not be feasible which inevitably precludes implementation. Contextual barriers are likely why pharmacological treatments are more readily available, as they are easier to standardise and deliver in routine care. According to the MS Trust database of self-identified Allied Health Professionals with expertise in MS, there are approximately 2,075 HCPs with an interest in MS, which indicates a limited number of allied HCPs to cater to the large population (n = 127,000).^31^ Therefore, to overcome the research-to-practice gap in this setting, focus is needed on integrating the available treatment evidence into a treatment that meets the demands of the context and can be integrated into service workflows. As there is limited HCP capacity, ways of delivery such as guided self-management or digital delivery should be considered. However, sensitivity to resource constraints should not come at the expense of appeal, sustainability, and long-term outcomes from the perspective of pwMS. To facilitate continued self-management, longer treatment duration and/or booster treatment sessions may be necessary, particularly given the progressive and unpredictable nature of MS, this may be more costly at the start, but likely cost-effective over time. Importantly, treatment guidelines need to clearly reflect the evidence, specifying which treatments are most/least effective to guide clinical practice. To sum up, a number of important considerations to address this research-to-practice are evident, including: 1) utilising the existing trial evidence in conjunction with sustainability from the perspective of both the NHS and pwMS, 2) working together with commissioners and policy makers from the outset, and 3) establishing a pathway from screening to treatment, including how a non-pharmacological treatment for fatigue is offered in consultations.

### Limitations

The strengths of this study are the large sample size and response rate. Limitations include possible recall bias since the mean number of years since diagnosis was 20 years and reliance on self-report. In order to more accurately capture fatigue treatments offered to pwMS and those ultimately received in routine care, it would be valuable to: 1) observe routine appointments, 2) conduct document analysis (e.g. appointment notes, referral forms), and 3) survey fatigue treatment experiences longitudinally among fatigued pwMS. The sample was predominantly White with a very limited representation of pwMS from BAME backgrounds and was restricted to only pwMS who could complete the survey in English; therefore, the findings are unlikely to generalise across diverse ethnic communities. Additionally, treatments actually received cannot be deciphered from what has been reported as offered by pwMS. It is also important to highlight the possibility of selection bias as the survey was administered through the UKMSR.

## Conclusion

Fatigue is among the most common and debilitating symptoms of MS. While the number of trials evaluating a variety of interventions for fatigue in MS continues to grow, this survey confirms that in the UK, fatigue is severely under-treated. Fewer than 10% have been offered an exercise or behavioural treatment for fatigue and the data suggest no standard or consistent NHS treatment for MS fatigue exists. Fatigue is associated with disability, disease progression, and quality of life, which in turn are associated with increased health care costs. Therefore, the value of improved fatigue management extends beyond the individual. To lead to tangible and lasting patient benefit, research focus needs to be redirected to understanding and acknowledging the context of delivery in intervention development and on the implementation of evidence-based approaches to treat fatigue.

## Supplemental Material

sj-docx-1-mso-10.1177_20552173211072274 - Supplemental material for Defining routine fatigue care in Multiple Sclerosis in the United Kingdom: What treatments are offered and who gets them? Click here for additional data file. Supplemental material, sj-docx-1-mso-10.1177_20552173211072274 for Defining routine fatigue care in Multiple Sclerosis in the United Kingdom: What treatments are offered and who gets them? by Federica Picariello, Jennifer Freeman and Rona Moss-Morris in Multiple Sclerosis Journal – Experimental, Translational and Clinical

sj-docx-2-mso-10.1177_20552173211072274 - Supplemental material for Defining routine fatigue care in Multiple Sclerosis in the United Kingdom: What treatments are offered and who gets them? Click here for additional data file. Supplemental material, sj-docx-2-mso-10.1177_20552173211072274 for Defining routine fatigue care in Multiple Sclerosis in the United Kingdom: What treatments are offered and who gets them? by Federica Picariello, Jennifer Freeman and Rona Moss-Morris in Multiple Sclerosis Journal – Experimental, Translational and Clinical

sj-docx-3-mso-10.1177_20552173211072274 - Supplemental material for Defining routine fatigue care in Multiple Sclerosis in the United Kingdom: What treatments are offered and who gets them? Click here for additional data file. Supplemental material, sj-docx-3-mso-10.1177_20552173211072274 for Defining routine fatigue care in Multiple Sclerosis in the United Kingdom: What treatments are offered and who gets them? by Federica Picariello, Jennifer Freeman and Rona Moss-Morris in Multiple Sclerosis Journal – Experimental, Translational and Clinical

sj-docx-4-mso-10.1177_20552173211072274 - Supplemental material for Defining routine fatigue care in Multiple Sclerosis in the United Kingdom: What treatments are offered and who gets them? Click here for additional data file. Supplemental material, sj-docx-4-mso-10.1177_20552173211072274 for Defining routine fatigue care in Multiple Sclerosis in the United Kingdom: What treatments are offered and who gets them? by Federica Picariello, Jennifer Freeman and Rona Moss-Morris in Multiple Sclerosis Journal – Experimental, Translational and Clinical

## Notes

1. A questionnaire was also sent out to NHS services that provide care to pwMS to gather (a) what resources and staff are available and (b) what fatigue treatments are routinely offered from the perspective of services. These findings will be reported separately.
