# Supplementary material for: Defining routine fatigue care in Multiple Sclerosis in the United Kingdom: What treatments are offered and who gets them?
Source: Mult Scler J Exp Transl Clin. 2022 Jan 20;8(1):20552173211072274. doi: 10.1177/20552173211072274 (PMC8796089; doi:10.1177/20552173211072274)
Supplement: sj-docx-1-mso-10.1177_20552173211072274 - Supplemental material for Defining routine fatigue care in Multiple Sclerosis in the United Kingdom: What treatments are offered and who gets them? [file sj-docx-1-mso-10.1177_20552173211072274.docx]

**Supplementary file A**

**Table A.1.** Core UKMSR outcomes.

| **Patient-reported outcome** | **Questionnaire** | **Further detail on questionnaire and scoring** |
| --- | --- | --- |
| Fatigue | Fatigue Severity Scale (FSS)^1^ | 9-item self-report measure of fatigue rated on a 7-point scale, from 1 (Strongly disagree) to 7 (Strongly agree).^1^ An average score of all items was calculated, with higher scores indicative of higher levels of fatigue and a cut-off of ≥ 5 considered to be indicative of experiencing severe levels of fatigue.^2^ The FSS was developed specifically for fatigue in MS^1^ and is widely used with good psychometric properties.^3^ In the current sample, Cronbach’s alpha was 0.95 suggestive of excellent internal consistency. |
| Distress | Hospital Anxiety and Depression Scale (HADS)^4^ | 14-item self-report measure of mood in patients with medical illnesses. Seven items relate to anxiety and seven relate to depression scored on a 4-point scale. Given the high correlation between depression and anxiety subscales, a total score that captures distress is more appropriate,^5^ with higher scores indicating greater levels of distress (score range 0–42). A review of the HADS found consistent support of its psychometric properties across samples.^6^ In this sample, Cronbach’s alpha was 0.92 suggestive of excellent internal consistency. |
| Walking ability | MS Walking Scale (MSWS)^7^ | 12-item self-report measure of perceptions of the impact of MS on walking ability over the past two weeks. In the first item, respondents are asked to indicate whether they are able to walk unassisted in any capacity. Only those who respond yes, proceed to the completion of the 12 items. The first three items are scored on a 3-point scale, ranging from 1 (Not at all) to 3 (A lot), and the remaining items are scored on a 5-point scale ranging from 1 (Not at all) to 5 (Extremely). A percentage score is calculated as the summed score relative to the highest possible score, with higher scores indicative of poorer self-reported walking ability. The MSWS is the most widely used and validated self-report measure of walking demonstrating good psychometric quality.^8^ In this sample, Cronbach’s alpha was 0.97 suggestive of excellent internal consistency. |
| Physical and psychological impact of MS | MS Impact Scale-29 (MSIS-29 version 2)^9^ | 29-item measure of the physical and psychological impact of MS over the past two weeks. Twenty items pertain to physical impact and 9 to psychological impact. Items are scored on a 5-point scale ranging from 1 (Not at all) to 4 (Extremely). A percentage score is calculated as the summed score relative to the highest possible score for overall physical impact of MS and one for overall psychological impact of MS score, with higher scores indicative of greater impact. The MSIS-29 is widely used and psychometrically rigorous.^10^ In this sample, Cronbach’s alpha was 0.97 for the overall scale suggestive of excellent internal consistency. |

1. Krupp LB, LaRocca NG, Muir-Nash J, Steinberg AD. The fatigue severity scale: Application to patients with multiple sclerosis and systemic lupus erythematosus. *Archives of Neurology.* 1989;46(10):1121-1123.

2. van Zanten JV, Douglas MR, Ntoumanis N. Fatigue and fluctuations in physical and psychological wellbeing in people with multiple sclerosis: A longitudinal study. *Multiple Sclerosis and Related Disorders.* 2021;47:102602.

3. Whitehead L. The measurement of fatigue in chronic illness: A systematic review of unidimensional and multidimensional fatigue measures. *Journal of Pain and Symptom Management.* 2009;37(1):107-128.

4. Zigmond AS, Snaith RP. The hospital anxiety and depression scale. *Acta Psychiatrica Scandinavica.* 1983;67(6):361-370.

5. Norton S, Cosco T, Doyle F, Done J, Sacker A. The Hospital Anxiety and Depression Scale: A meta confirmatory factor analysis. *Journal of Psychosomatic Research.* 2013;74(1):74-81.

6. Bjelland I, Dahl AA, Haug TT, Neckelmann D. The validity of the Hospital Anxiety and Depression Scale: an updated literature review. *Journal of Psychosomatic Research.* 2002;52(2):69-77.

7. Hobart J, Riazi A, Lamping D, Fitzpatrick R, Thompson A. Measuring the impact of MS on walking ability: The 12-Item MS Walking Scale (MSWS-12). *Neurology.* 2003;60(1):31-36.

8. McGuigan C, Hutchinson M. Confirming the validity and responsiveness of the Multiple Sclerosis Walking Scale-12 (MSWS-12). *Neurology.* 2004;62(11):2103-2105.

9. Hobart J, Lamping D, Fitzpatrick R, Riazi A, Thompson A. The multiple sclerosis impact scale (MSIS-29) a new patient-based outcome measure. *Brain.* 2001;124(5):962-973.

10. Khurana V, Sharma H, Afroz N, Callan A, Medin J. Patient‐reported outcomes in multiple sclerosis: A systematic comparison of available measures. *European Journal of Neurology.* 2017;24(9):1099-1107.
