# Supplementary material for: Defining routine fatigue care in Multiple Sclerosis in the United Kingdom: What treatments are offered and who gets them?
Source: Mult Scler J Exp Transl Clin. 2022 Jan 20;8(1):20552173211072274. doi: 10.1177/20552173211072274 (PMC8796089; doi:10.1177/20552173211072274)
Supplement: sj-docx-2-mso-10.1177_20552173211072274 - Supplemental material for Defining routine fatigue care in Multiple Sclerosis in the United Kingdom: What treatments are offered and who gets them? [file sj-docx-2-mso-10.1177_20552173211072274.docx]

**Supplementary file B**

**Table B.1.** Overview of content covered in the fatigue treatments offered questionnaire.

| Items | Responding format |
| --- | --- |
| Treatments | Response options |
| Medications for fatigue | - None - Amantadine (Symmetrel or Lysovir) - Modafinil (Provigil) - Prokarin (a skin patch that contains caffeine and histamine) - Other prescription medications [free text box] - Over the counter medications/supplements (such as liquid iron, beetroot powder, vitamin B12) [free text box] - I do not recall the name of the medication |
| Non-pharmacological treatments:   - Exercise - Behavioural therapy - Dietitian support - Nurse support - Occupational therapy support - Social care support | - No - Yes, I have but not for my fatigue - Yes, I have for my fatigue specifically   The two yes response options were included to maximise certainty that treatments reported are specific for fatigue.  For dietitian support, nurse support, occupational therapy support, and social care support, respondents were asked to provide further detail on what that consisted of in a free text format. |
| Exercise | - Aerobic (exercise that causes you to be quickly out of breath, like running, swimming, walking, hiking) - Resistance (exercise that improves muscular strength and endurance, like squats, bicep curls) - Yoga - Balance (exercises that improve your ability to control and stabilize your body's position) - Physiotherapy - Other exercise [free text box] |
| Behavioural therapy | - Counselling (talking treatment that involves counsellor listening and helping to find solutions to cope with the issues you face) - CBT (cognitive behavioural therapy - talking treatment which focuses on how your thoughts affect your feelings and behaviour) - Mindfulness (talking treatment that includes meditation and breathing exercises) - FACETS (Fatigue: Applying Cognitive behavioural and Energy effectiveness Techniques to lifestyle - six group-sessions focused on self-management of fatigue) - Not sure [free text box] - Other psychological approach [free text box] |
| Respondents were asked to rate perceived improvement in fatigue following each type of treatment on a scale | From 1 (Very much better) to 7 (Very much worse), with higher scores indicative of deterioration in fatigue. |
| Respondents were asked whether they would like better treatment provision for fatigue | - Yes - No |
