# Supplementary material for: Defining routine fatigue care in Multiple Sclerosis in the United Kingdom: What treatments are offered and who gets them?
Source: Mult Scler J Exp Transl Clin. 2022 Jan 20;8(1):20552173211072274. doi: 10.1177/20552173211072274 (PMC8796089; doi:10.1177/20552173211072274)
Supplement: sj-docx-3-mso-10.1177_20552173211072274 - Supplemental material for Defining routine fatigue care in Multiple Sclerosis in the United Kingdom: What treatments are offered and who gets them? [file sj-docx-3-mso-10.1177_20552173211072274.docx]

**Supplementary file C**

**Table C.1.** Differences between respondents reporting fatigue and those not reporting fatigue based on sociodemographic, MS-related, and psychological variables.

| Variables | Experiencing fatigue (3,943*) | Not experiencing fatigue (424*) | Comparison statistic |
| --- | --- | --- | --- |
| Age M (SD) | 55.16 (SD=11.22) | 54.80 (SD=11.95) | t(506.59)=-0.59, p=0.558 |
| Gender N (%) | Female 2930 (74.3%)  Male 1010 (25.6%) | Female 312 (73.6%)  Male 112 (26.4%) | χ^2^=0.12, p=0.272 |
| Ethnicity N (%) | White 3678 (93.7%)  Black 24 (0.6%)  Asian 38 (1.0%)  Mixed 23 (0.6%)  Other 162 (4.1%) | White 388 (91.5%)  Black 6 (1.4%)  Asian 8 (1.9%)  Mixed 4 (0.9%)  Other 18 (4.2%) | Fischer’s exact test=7.83, p=0.084 |
| Education status N (%) | School 766 (19.4%)  Occupational certificate/diploma 1210 (30.7%)  Undergraduate degree 1016 (25.8%)  Postgraduate degree 727 (18.4%)  Other 224 (5.7%) | School 69 (16.3%)  Occupational certificate/diploma 108 (25.5%)  Undergraduate degree 115 (27.1%)  Postgraduate degree 106 (25.0%)  Other 26 (6.1%) | χ^2^=14.49, p=0.006 |
| Employment status N (%) | Regular paid employment 1347 (34.2%)  Self-employed 245 (6.2%)  Engaged in voluntary work 50 (1.3%)  In formal education 22 (0.6%)  Looking after home/family 115 (2.9%)  Retired 1216 (30.8%)  Unemployed 88 (2.2%)  Temporarily sick/disabled 50 (1.3%)  Permanently sick/disabled 755 (19.2%)  Other reasons not working 38 (1.0%)  Not applicable 16 (0.4%) | Regular paid employment 205 (48.3%)  Self-employed 32 (7.5%)  Engaged in voluntary work 5 (1.2%)  In formal education 2 (0.5%)  Looking after home/family 12 (2.8%)  Retired 137 (32.3%)  Unemployed 6 (1.4%)  Temporarily sick/disabled 1 (0.2%)  Permanently sick/disabled 21 (5.0%)  Other reasons not working 2 (0.5%)  Not applicable 1 (0.2%) | χ^2^=72.48, p<0.001 |
| MS subtype N (%) | RRMS 2186 (55.4%)  SPMS 1045 (26.5%)  PPMS 470 (11.9%)  Benign 75 (1.9%)  Unknown 167 (4.2%) | RRMS 268 (63.2%)  SPMS 64 (15.1%)  PPMS 51 (12.0%)  Benign 21 (5.0%)  Unknown 20 (4.7%) | χ^2^=40.15, p<0.001 |
| Time since MS diagnosis in years M (SD) | 19.38 (SD=11.80) | 17.27 (SD=12.01) | t(4160)=-3.41, p=0.001 |
| Web-based EDSS M (SD) | 5.18 (SD=1.97) | 3.26 (SD=2.24) | t(917)=-8.09, p<0.001 |
| Fatigue severity (FSS average score) M (SD) | 5.07 (SD=1.34) | 2.56 (SD=1.18) | t(521.799)=-40.28, p<0.001 |
| Walking impairment (MSWS) M (SD) | 41.47 (SD=31.55) | 19.20 (SD=26.08) | t(457.174)=-14.27, p<0.001 |
| Walking ability (MSWS) N (%) | Cannot walk unassisted 1156 (30.3%)  Can walk unassisted 2664 (69.7%) | Cannot walk unassisted 79 (19.3%)  Can walk unassisted 330 (80.7%) | χ^2^=21.41, p<0.001 |
| Distress (HADS) M (SD) | 13.94 (SD=8.18) | 7.56 (SD=6.65) | t(558.504)=-18.09, p<0.001 |
| MS Impact – Physical (MSIS-P) M (SD) | 33.82 (SD=19.81) | 16.08 (SD=17.46) | t(535.205)=-19.36, p<0.001 |
| MS Impact – Mental (MSIS-M) M (SD) | 29.88 (SD=17.90) | 12.09 (SD=12.28) | t(621.788)=-26.57, p<0.001 |

*Some missingness may be present depending on data availability for each sociodemographic, MS-related, and psychological characteristic used for comparison.
