# Supplementary material for: Defining routine fatigue care in Multiple Sclerosis in the United Kingdom: What treatments are offered and who gets them?
Source: Mult Scler J Exp Transl Clin. 2022 Jan 20;8(1):20552173211072274. doi: 10.1177/20552173211072274 (PMC8796089; doi:10.1177/20552173211072274)
Supplement: sj-docx-4-mso-10.1177_20552173211072274 - Supplemental material for Defining routine fatigue care in Multiple Sclerosis in the United Kingdom: What treatments are offered and who gets them? [file sj-docx-4-mso-10.1177_20552173211072274.docx]

**Supplementary file D**

**Table D.1.** Perceived change in fatigue following other (not exercise or behavioural therapy) non-pharmacological fatigue treatments received.

|  | Perceived change in fatigue Median (Interquartile range, range) | Frequency of response options selected | |
| --- | --- | --- | --- |
| Dietitian support | 4 (interquartile range=., range=3-4) | Better or very much better  A little better  No change  A little worse  Worse or very much worse | 0  1 (33.3%)  2 (66.7%)  0  0 |
| Nurse support | 3 (interquartile range=1, range=1-5) | Better or very much better  A little better  No change  A little worse  Worse or very much worse | 22 (15.2%)  65 (44.8%)  55 (37.9%)  3 (2.1%)  0 |
| Social care support | 3 (interquartile range=2, range=2-4) | Better or very much better  A little better  No change  A little worse  Worse or very much worse | 7 (29.2%)  10 (41.7%)  7 (29.2%)  0  0 |
| Occupational therapy support | 3 (interquartile range=0, range=1-5) | Better or very much better  A little better  No change  A little worse  Worse or very much worse | 23 (18.9%)  71 (58.2%)  27 (22.1%)  1 (0.8%)  0 |
